# A Passive Exoskeleton Can Push Your Life Up: Application on Multiple Sclerosis Patients

**DOI:** 10.1371/journal.pone.0077348

**Published:** 2013-10-25

**Authors:** Francesco Di Russo, Marika Berchicci, Rinaldo Livio Perri, Francesca Romana Ripani, Maurizio Ripani

**Affiliations:** 1 Department of Human Movement, Social and Health Sciences, University of Rome “Foro Italico”, Rome, Italy; 2 Neuropsychology Unit, IRCCS Santa Lucia Foundation, Rome, Italy; 3 Department of Anatomical, Histological, Forensic Medicine and Locomotor Sciences, University of Rome “La Sapienza”, Rome, Italy; University of Bologna, Italy

## Abstract

In the present study, we report the benefits of a passive and fully articulated exoskeleton on multiple sclerosis patients by means of behavioral and electrophysiological measures, paying particular attention to the prefrontal cortex activity. Multiple sclerosis is a neurological condition characterized by lesions of the myelin sheaths that encapsulate the neurons of the brain, spine and optic nerve, and it causes transient or progressive symptoms and impairments in gait and posture. Up to 50% of multiple sclerosis patients require walking aids and 10% are wheelchair-bound 15 years following the initial diagnosis. We tested the ability of a new orthosis, the “Human Body Posturizer”, designed to improve the structural and functional symmetry of the body through proprioception, in multiple sclerosis patients. We observed that a single Human Body Posturizer application improved mobility, ambulation and response accuracy, in all of the tested patients. Most importantly, we associated these clinical observations and behavioral effects to changes in brain activity, particularly in the prefrontal cortex.

## Introduction

**Figure 1:**
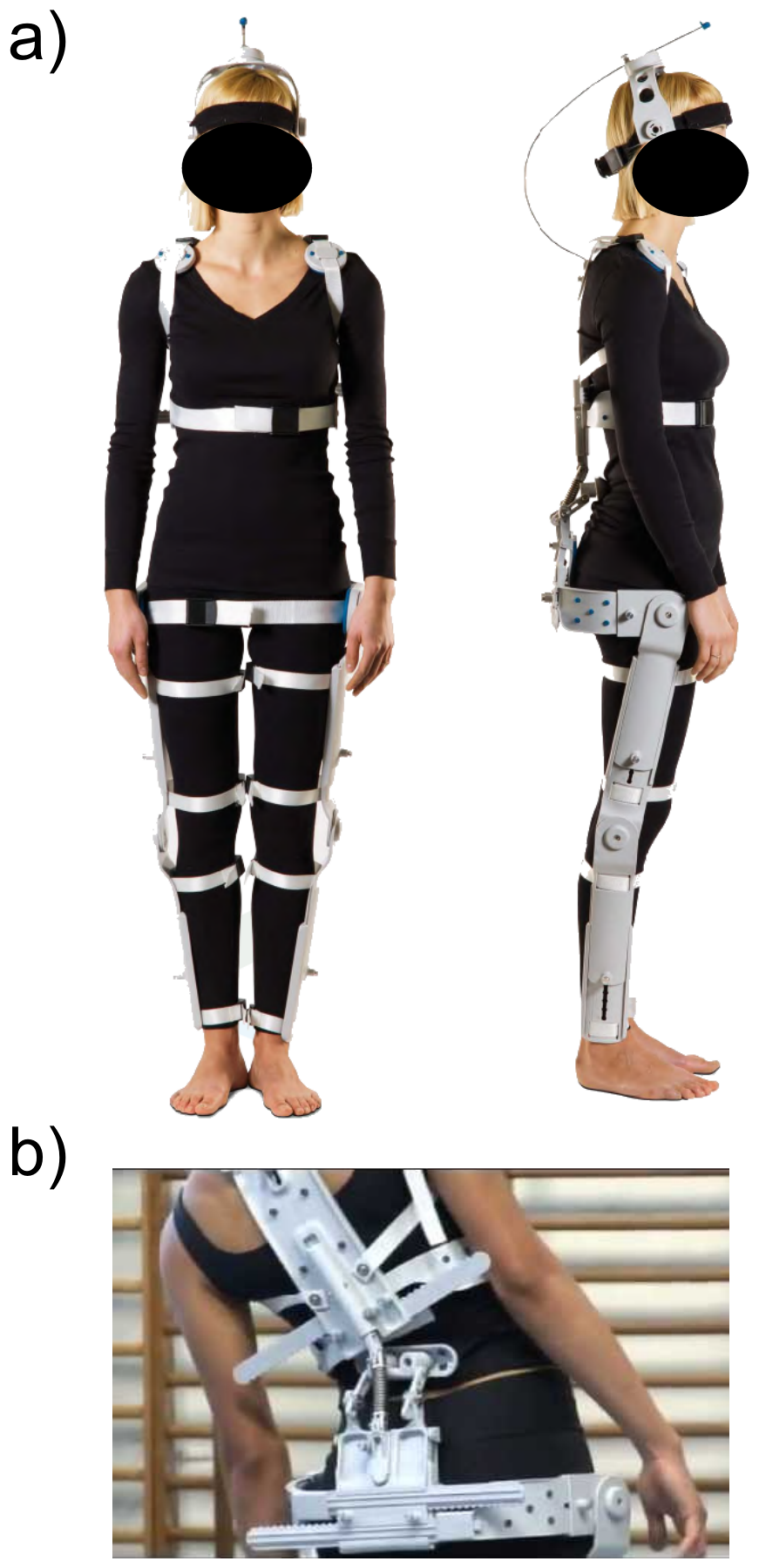
The Human Body Posturizer (HBP) orthosis. a) Frontal and lateral view of the HBP exoskeleton worn by an actor. b) Close-up of the rear view showing the articulation capacities. The subject of the photograph has given written informed consent, as outlined in the PLOS consent form, to publication of their photograph.

Human exoskeletons are used for enhancing people's strength, endurance and speed in many activities, and they have recently been shown to improve the quality of life in people with disabilities [1]. Most exoskeletons constructed for patients are very expensive and cumbersome, partially due to being motorized and computerized [2], [3]. The “Human Body Posturizer” (HBP) system is much cheaper, lighter and compact (e.g., portable in a case) than other commercially available orthoses. The key concept of this innovative system is based on its fully mobile and mechanically passive components that can be adapted to one's body shape, enabling users to assume a more physiological posture. The HBP orthosis is a non-powered, modular and flexible exoskeleton. It is fully articulated and made of solid plastic and metal that extends from the head to the lower limbs, consisting of four separated modules (Figure 1a, b).

The idea is that the HBP acts on proprioceptive receptors by transmitting signals on the correct posture to supra-axial nerve centers, which are integrated and interpreted in the central nervous system. Previous studies [4], [5] reported effects of the HBP on postural dynamics. These studies found that the HBP may increase the degree of symmetry in trunk and lumbar regions of the spinal column and also reduce the risk of falling in the elderly. In the present study, we investigated the effect of the HBP on multiple sclerosis (MS) patients. MS is a neurological condition characterized by lesions of the myelin sheaths that encapsulate the neurons of the brain, spine and optic nerve, and it causes transient or progressive symptoms and impairments in gait and posture [6]. Up to 50% of MS patients require walking aids and 10% are wheelchair-bound 15 years following the initial diagnosis [7]. To investigate the effects of the HBP on movement control in MS patients with postural and locomotor deficits, we studied the brain correlates of motor planning and response execution during a discriminative visuo-motor task. The motor preparation in those types of complex tasks relies not only on the engagement of the premotor cortex and motor areas, but alsoon the parietal areas, the orbitomedial and the dorsolateral prefrontal cortex [8], [9], [10], [11], [12]. The participation of the prefrontal cortex (PFC) on motor preparation and execution has been interpreted as a compensatory activity improving response accuracy in the control of complex or difficult actions. In older adults, PFC control becomes progressively stronger and is associated with a slower response time, though response accuracy remains high [13]. Furthermore, a physically active lifestyle appears to counteract PFC over-recruitment during action preparation and to preserve fast response times, especially after 35–40 years of age [12]. The PFC is the highest stage of neural integration in the perception-action cycle, which allow us successive interaction with the environment in the goals pursuit, playing also a critical role in working memory, future planning and action monitoring. Once the goal is reached, the PFC sends feedback to posterior associative areasIn other words, the PFC coordinates cognitive functions in the temporal organization of behavior, that is the creation of coherent behavioral sequences toward the goal. All the prefrontal functions might be defined pre-adaptive, because they pre-adapt the individual to a future predicted environment [14].

The effects of the HBP treatment on MS patients were tested using behavioral and neurophysiological measures. The PFC activity of the participants was studied using a high-resolution electroencephalographic (EEG) recording by analyzing both movement-related cortical potentials (MRCPs) and event-related potentials (ERPs) during a Go/No-go task, which is a visuo-motor discriminative response task that is widely used to assess executive functions.The main hypothesis is based on clinical observations(made by the authors MR and FRR) that a single HBP treatment was already effective in improving the control of bipedal standing posture and gait in MS patients via corrective signals arising from the body.We expected to observe changes in electro-cortical activity (especially PFC functions) given that thestimulation of the HBP could make more resources available for motor control. To the best of our knowledge, this study represents the first investigation of the effects of orthesis stimulation on the executive functions mediated by the PFC in MS patients.

## Materials and Methods

### Ethics Statement

After a full explanation of the procedures, all subjects provided their written informed consent prior the experimentaccording to the Declaration of Helsinki. The study and all of the procedures were approved by the IRCSS Santa Lucia Foundation of Rome ethics committee.

### Participants

We examined six patients with relapsing-remitting MS type (five females, 33–56 years old; mean 47.3 yrs) and six healthy controls that were gender-, education- and age-matched (five females, 32–57 years old; mean 46.7 yrs) to the patient group. MS patients with moderate to severe functional disabilities (including ambulation impairment) were selected based on the expanded disability status scale (EDSS score 5–8) [15]. All patients had multiple lesions in the white matter, cortical and subcortical areas, but no reported lesions in the PFC. The clinical data of the patients are reported in Table 1. All participants had normal and/or corrected vision and were fully right-handed (Edinburgh handedness inventory) [16].

**Table 1:** Demographic and clinical data of the patients.

Patient #	Age (yrs)	Gender	TFO (yrs)	Education (yrs)	Brain lesions	EDSS Pre-session	EDSS Post-session
1	33	F	21	18	F, P	6.5	5.5
2	43	F	13	13	F, P	7.5	7.0
3	45	F	5	13	T, P	5.5	5.0
4	49	F	15	13	T, P	8.0	7.5
5	55	M	22	18	F, T, P	6.0	5.5
6	56	F	14	13	T, P	5.5	5.5

TFO = Time from onset (years of disease duration). Brain lesions: F = Frontal, T = Temporal, P = Parietal.

### Description of the exoskeleton

The HBP is made of four separated modules (Figure 1a, b). The cranial-cervical module is a helmet with a cervical spring mechanism connected to the dorsal module, which enables complex head and neck movements and stimulates the attitude of the cervical trait. The dorsal module rests on the back and is the central HBP element. It has chest straps that can be adjusted and secured to the shoulders with braces. The lumbar-sacral module is positioned at the center of the sacrum; it is articulated with the dorsal element (Figure 1b) and secured with straps. This module has an adjustable support that allows modulating a forward thrust to the lumbar region, which applies high proprioceptive stimuli to the hips and pelvis. The lower limbs module has a lateral pelvic support at the level of the hip joint and is connected to each leg using two lateral brackets at the level of the thighs and legs. The total weight of the HBP is approximately 2.2 Kg.

### Task

For the EEG measures, each participant was tested in a sound-attenuated, dimly lit room after a 64-channel EEG cap was mounted on the scalp. Visual stimuli (i.e., four squared configurations made by vertical and horizontal bars) were randomly displayed for 260 ms with equal probability (*p* = 0.25); the stimulus-onset asynchrony varied from 1 to 2 s to avoid time prediction effects on the RTs (for more details on the paradigm, see 14). The participants performed a discriminative response task, which was derived from a widely used clinical test [17]. The participants had to press a button with the right hand when a target appeared on the screen (Go stimuli; p = 0.5) and withhold the response when a non-target appeared (No-Go stimuli; p = 0.5).

**Figure 2:**
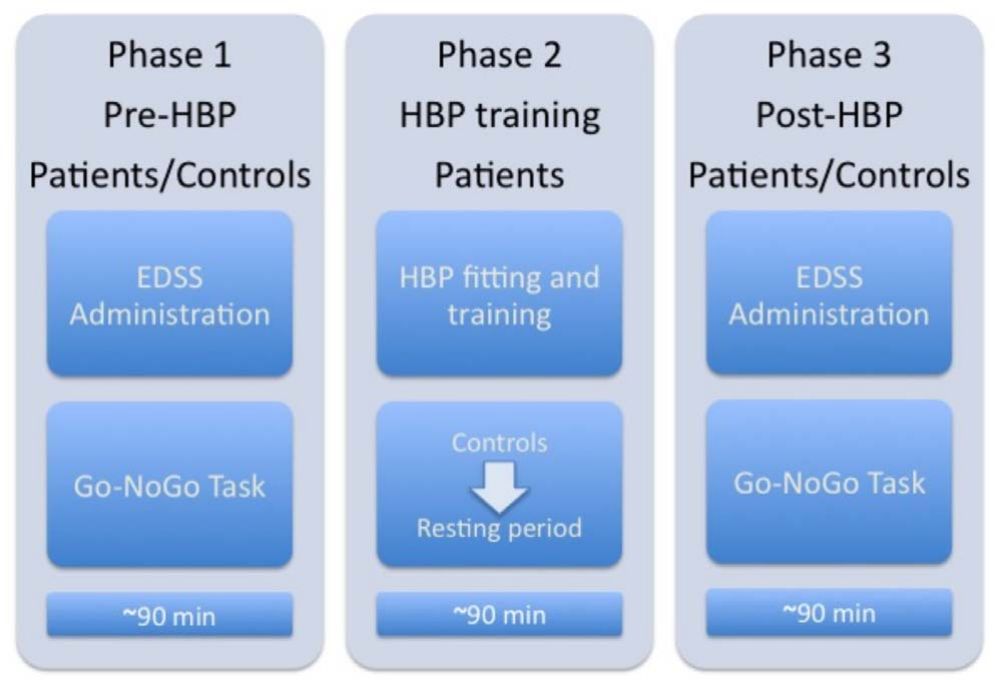
Chart-flow of the experimental protocol. EDSS refers to Expanded Disability Status Scale; HBP refers to Human Body Posturizer; Go-NoGo task includes both EEG and behavioral recordings.

Behavioral and EEG measurements were recorded during the Go/No-Go task within a single day in two identical sessions: pre- and post-HBP treatment. After the pre-HBP session recordings, the HBP was mounted and finely calibrated to the patient's body. Patients wore the HBP for 60 minutes and were asked to freely move and walk to the best of their abilities. The HBP was later removed and post-session measurements were performed. The EDSS was fully administered before and after the HBP treatment, focusing on the ambulation. The total duration of the experiment was approximately 4.5 hours. To control for the possible learning effects of repeating the same task, healthy controls executed the same tasks as the patients (except for the EDSS) with identical timing. However, controls did not wear the HBP, in order to isolate possible learning effects from the HBP effects (which could be the topic of future studies). A diagram representing the experimental procedure is reportedin Figure 2.

### Behavioral recording and analysis

In the discriminative response task, the median RTs for the correct trials were calculated, and accuracy was measured by the percentage of false alarms (i.e., responses to No-Go stimuli). The EDSS was administered in MS patients before the experimental session, within the clinical assessment (pre-session) and after the post-session. Ambulation performance (walking distance) was assessed indoors on a linear 50 m hallway, which was retraced every time a patient was able to walk. To obtain an EDSS score of 5.0 (the best score in the present group), patients had to walk without aid or rest for at least 200 m. During and after the HBP training, the patients were asked to report any mental and physical sensation about their self, in particular the effects of the HBP on their posture and gait. The authors MR and FRR clinically evaluated the patients before and after the treatment to note changes in their balance and ambulation capability. No instrumental examination of posture, balance and/or walking could be performed due to time constrains when studying suffering patients.

### Electrophysiological recording and analysis

EEGs were recorded using the BrainVision™ system (BrainProducts GmbH., Munich, Germany) with 64 electrodes mounted according to the 10-10 International System and were initially referenced to the left mastoid. The EEGs were digitized at 250 Hz, amplified (bandpass of 0.01–80 Hz, including a 50 Hz notch filter) and stored off-line for averaging. The electrophysiological data were re-referenced to average mastoids, and computerized artifact rejection was performed prior to signal averaging to discard epochs contaminated by artifacts. Horizontal eye movements were monitored with a bipolar recording from electrodes at the left and right outer canthi. The blinks and vertical eye movements were recorded with an electrode below the left eye, which was referenced to site Fp1. Trials with artifacts (e.g., blinks or gross movements) were automatically excluded from averaging, and eye movement artifacts were reduced throughout with the Gratton algorithm [18].

**Figure 3:**
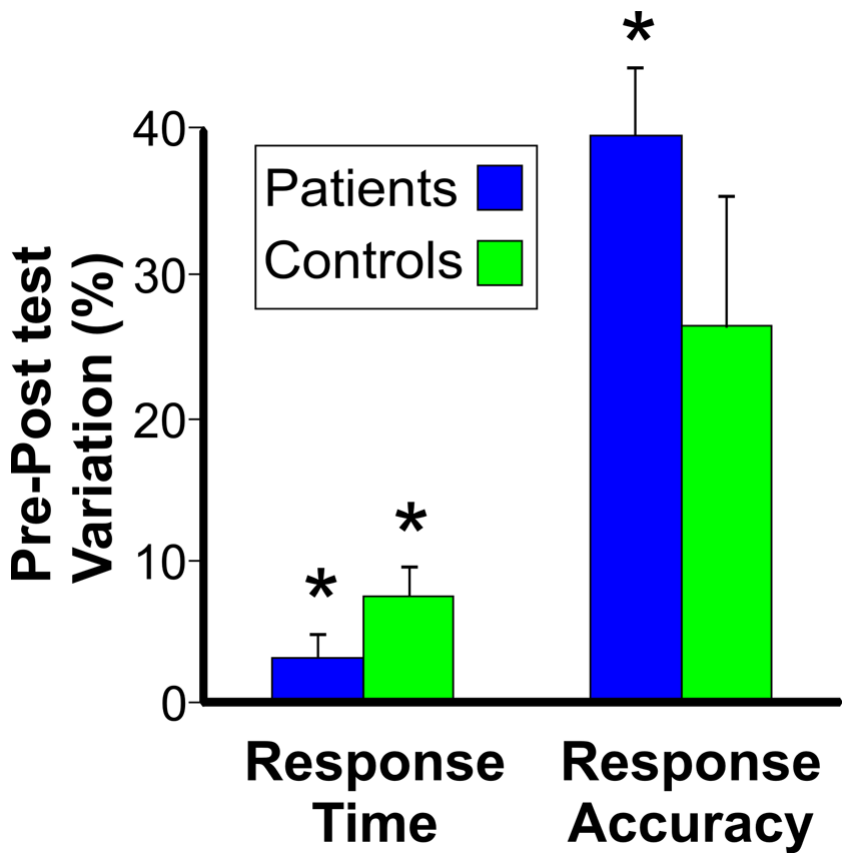
Behavioral results (response time and false alarms) in the two groups. Data are expressed as the percentage variation between the pre- and post-sessions. Stars indicate statistically significant variations.

MRCPs were segmented and averaged into non-overlapping epochs of 2000 ms, 1500 ms before and 500 ms after response onset, which was defined as time zero. The baseline was defined by the mean voltage during the initial 300 ms of the averaged epochs. To further reduce high-frequency noise, the group-averaged MRCPs were low-pass filtered (i.e., Butterworth) at 15 Hz. MRCP onset latency and mean amplitude of the pre-motor activity from 1000 to 500 ms before the response onset were measured. Amplitude measurements were taken at the peak electrode on the basis of the scalp voltage distribution of the topographical maps (see Figure 3) within the prefrontal lobe (Fp2 in both groups) and the motor and premotor areas (Cz).

The stimulus-locked ERPs were segmented and averaged into non-overlapping epochs of 1100 ms, 100 ms before and 1000 ms after movement onset, which was defined as time zero. Peak latency and amplitude of the typical ERP components (P1, N1, N2 and P3) were measured at the peak electrode (see 14 for more details).

### Statistical analysis

The EDSS scores were subjected to repeated measures ANOVA (pre- vs. post-session). All other behavioral and electrophysiological parameters mentioned above were separately subjected to a factorial (2x2) ANOVA using group (patients vs. controls) and session (pre vs. post). Post-hoc comparisons were performed using the Tukey HSD test. The overall alpha level was fixed at 0.05 after the Geisser-Greenhouse correction.

### Source Analysis

The intracranial sources of prefrontal activity in the MRCP were determined using the different approaches of the BESA 2000 system. We first used the spatiotemporal source analysis module of the BESA that estimates the location, orientation and time course of multiple equivalent dipolar sources by calculating the scalp distribution obtained for a given model (forward solution). This distribution was then compared to that of the actual MRCP. Interactive changes in the source location and orientation led to minimization of the residual variance between the model and the observed spatiotemporal MRCP distribution. The three-dimensional coordinates of each dipole in the BESA model were determined with respect to the Talairach axes. The possibility of interacting dipoles was reduced by selecting solutions with relatively low dipole moments with the aid of an “energy” constraint (weighted 20% in the compound cost function, as opposed to 80% for the residual variance). The optimal set of parameters was found in an iterative manner by searching for a minimum in the compound cost function. Latency range for fitting was from −1000 to −500 ms. As a second approach, the noise-normalized minimum-norm method was employed to estimate the current density dipoles on the cortical scalp. The minimum-norm estimation (MNE) approach is a method regularly used to estimate the distributed electrical current in a brain image at each time sample. MNE is able to resolve the inverse problem without a priori constraints and reveal the unique constellation of current elements that models the recorded electric field distribution with the smallest amount of overall current [19]. The employed algorithm minimizes the source vector current derived from 1,426 evenly distributed dipoles located 10% and 30% below the surface of the brain by using the approach adopted by Dale and Sereno [20]. To improve the MNE, we also included depth-weighting parameters across the entire source space because depth-weighted MNEs can improve the spatial accuracy by allowing displacement errors within 12 mm [21]. Finally, the Multiple Source Beamformer (MSBS) approach was used. The BESA beamformer is a modified version of the linearly constrained minimum variance vector beamformer in the time-frequency domain. It allows one to image evoked and induced oscillatory activity in a user-defined time-frequency range, where time is taken relative to a triggered event. A beamformer operator is designed to pass signals from the brain region of interest without attenuation while minimizing interference from activity in all other brain regions. Traditional single-source beamformers are known to mislocalize sources if several brain regions have highly correlated activity. Therefore, the BESA beamformer extends the traditional single-source beamformer to implicitly suppress activity from possibly correlated brain regions. This is achieved by using a multiple source beamformer calculation that contains not only the lead-fields of the source at the location of interest but also those of possibly interfering sources. As a default, BESA uses a bilateral beamformer, where contributions specifically from the homologue source in the opposite hemisphere are taken into account. This allows for imaging of highly correlated bilateral activity in the two hemispheres that commonly occurs during processing of external stimuli. In addition, the beamformer computation can take into account possibly correlated sources at arbitrary locations that are specified in the current solution. In every approach, the BESA assumed a realistic approximation of the head (based on the MRI of 24 subjects).

## Results

### Behavioral data

Behaviorally, the patients were much slower (p = 0.0049) than the controls (555 ms and 456 ms response times, respectively). In the post-session, the response time was reduced in both patients and controls (p = 0.0016), but this effect was twice as strong in controls (3.2% and 7.4% response time reduction, respectively), likely indicating a task-learning effect.In the post-session, false alarms (indicating response accuracy) were significantly reduced in the patients (7.8% change, p = 0.0019), but not in the controls (2.5% change), with no significant differences between groups. Regarding the response time, ANOVA showed significant effects of group (F_1,10_ = 12.9, p<0.0049, η^2^p = 0.56) and session (F_1,10_ = 18.4, p<0.0016, η^2^p = 0.65). The interaction was not significant (F_1,10_ = 2.4, p<0.1498, η^2^p = 0.19).

Regarding the response accuracy (false alarms), ANOVA showed significant effects of session (F_1,10_ = 17.7, p<0.0018, η^2^p = 0.64) and interaction (F_1,10_ = 10.2, p<0.0096, η^2^p = 0.50). The effect of group was not significant (F_1,10_ = 1.5, p<0.2448, η^2^p = 0.13). Post-hoc analysis on the significant interaction showed that the accuracy in the post-session increased (p = 0.019) in the patients group, but not in the controls group. Figure 3 represents the significant pre-and post-test variations in both response time and accuracy in the two groups.

The ambulation and standing posture capabilities of the patients visibly improved. The clinical evaluation highlighteda better trunk alignment and more balanced posture during walkingboth during and after the HBP training. Patients self-report unanimously indicated that the main sensation related to the HBP was increased body lightness and balance. Most of the patients were able to walk quickly and some actually did run. All of the patients reported to be able to do thinks, as speeded walking and running, while wearing the HBP that they did not before (see Table 1). The EDSS score was significantly reduced (F_1,5_ = 15.0, p = 0.0117) by approximately 0.5 points. The patients showed a more stable posture and were able to walk a greater distance (measured in meters) than before the treatment.For instance, the EDSS of patient #6 (i.e. the oldest) did not change, whereas it reached one point in patient #1 (i.e. the youngest).

### Electrophysiological data

**Figure 4:**
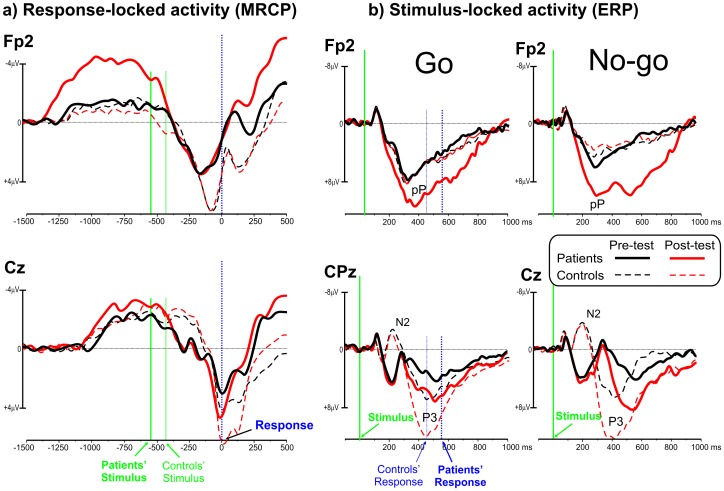
Grand average of the movement- and event-related potentials waveforms. a) MRCP waveforms (response-related activity) over the prefrontal lobe (Fp2) and the sensorimotor cortex (Cz). Time zero represents the response onset. Vertical green lines represent the median time of stimulus onset in the two groups. b) ERP waveforms (stimulus-related activity) over the prefrontal lobe (Fp2) and the sensorimotor cortex for both Go (CPz) and No-go (Cz) stimuli. Time zero represents the stimulus onset. Vertical blue lines represent the median time of response onset in the two groups.

Figure 4a shows the brain activity before the action and stimulus onset. The MRCPs of the patients did not differ from the controls in the pre-session, but they consistently increased (by a factor of 3) during the post-sessionover the PFC (p = 0.0007) and, to a lesser degree (50%), over the premotor areas (p = 0.063, ns). In the controls, no significant MRCP changes were detected (p = 0.24, ns). With respect to the pre-movement MRCP over the PFC, ANOVA showed significant effects of session (F_1,10_ = 12.1, p<0.0059, η^2^p = 0.42), group (F_1,10_>8.7, p<0.0145, η^2^p = 0.37) and interaction (F_1,10_ = 23.8, p<0.0006, η^2^p = 0.59). The post-hoc tests on the interaction showed that in the post-session the PFC activity of the patients increased (p = 0.0007), but did not change in controls. The PFC activity of patients was similar to controls in the pre-session, but was larger than controls in the post-session (p = 0.0006). Regarding the pre-movement MRCP over the premotor areas, ANOVA did not show significant effects.

The ERPs (Figure 4b) showed a consistent delay in the patients' brain responses compared to the controls. In the patients, the N2 and the P3 components (reflecting response inhibition and monitoring, respectively; [22]) were much smaller than the controls for both the Go and No-go stimuli (p<0.0082). In both groups, the N2 and P3 componentschanged between sessions. With respect to stimulus-locked ERPs, ANOVA on the visual P1 and N1 components showed a significant effect of group (F_1,10_>10.2, p<0.0096, η^2^p>0.47) on the peak latency for both Go and No-go stimuli. Latencies were slower in the patients (P1 = 125 ms, N1 = 175 ms) than controls (P1 = 105 ms, N1 = 155 ms). No effects of session and interaction were found. ANOVA on the P1 and N1 amplitude did not show significant effects, though the P1 was smaller in the patients. Regarding the N2 and P3 latencies, the effects of group were significant (F_1,10_>14.7, p<0.0033, η^2^p>0.55) for both Go and No-go stimuli (patients: N2 = 305 ms, P3 = 520 ms; controls: N2 = 210 ms, P3 =  440 ms). The effects of session were also significant (F_1,10_>11.3, p<0.0072, η^2^p>0.49) for both Go and No-go stimuli. Similarly, for the N2 and P3 amplitude, the effects of group were significant (F_1,10_>10.8, p<0.0082, η^2^p>0.37) for both Go and No-go stimuli, with smaller amplitudes in the patients than controls. The effects of session were also significant (F_1,10_>13.4, p<0.0044, η^2^p>0.47) for both Go and No-go stimuli. The interaction was not significant.

In the pre-session, the pP component (representing PFC compensatory activity for executive motor control between 300 and 800 ms following stimulus onset; 14) did not differ between groups. In the patients, the pP was larger in the post-HBP session than in the pre-HBP session (p = 0.0004). The P3 activity (peaking at 600 ms) was larger and started earlier in the post-HBP session than in the pre-HBP session (p<0.0012). These effects were stronger for the No-go stimuli. With respect to the pP component, ANOVA showed significant effects of session (F_1,10_ = 8.6, p<0.0150, η^2^p = 0.22), group (F_1,10_>8.9, p<0.0137, η^2^p = 0.31) and interaction (F_1,10_ = 29.4, p<0.0003, η^2^p = 0.61). The post-hoc tests on the interaction showed that in patients, the pP increased in the post-session (p = 0.0004), while it did not change in controls. The pP of patients was similar to controls in the pre-session, but was larger than controls in the post-session (p = 0.0003).

In the control subjects, the pP in the pre- and post-sessions did not significantly differ, and the P3 was much larger (p = 0.0051) in the post-session thanin the pre-session. These results indicate that while the P3 effect might be attributed to task learning, the effect on the pP might be ascribed to the HBP treatment.

### Scalp topography and source localization

Scalp topography and source localization of the MRCPs confirmed that the prefrontal activity arose bilaterally from Brodmann area 10 in the PFC (prefrontal pole). Analysis of the MRCP source was employed using three independent techniques (spatiotemporal, minimum-norm and multiple source beamformer), all of which yielded consistent results.

**Figure 5:**
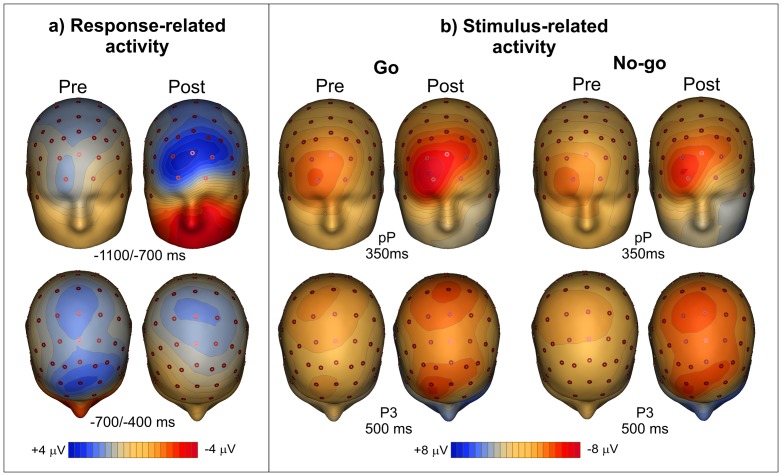
Scalp topography maps. Scalp topography of response-related (a) and stimulus-related (b) activity in MS patients in the pre- and post-session recordings. The scalp distribution of the pre-motor and post-stimulus activity on PFC is very similar except for the polarity.

Figure 5a shows the scalp topography during earlier (−1100/−700 ms) and later (−700/−400 ms) motor preparation of patients. In the earlier period, the HBP treatment dramatically increased the prefrontal negativity. In the later period, the activity over premotor and motor cortex did not change. Figure 5b shows the scalp topography of the stimulus-related activity with Go and No-go stimuli. Both pP and P3 waves were enhanced in the post-session even though the pP increase was much larger, especially for target (Go) stimuli.

**Figure 6:**
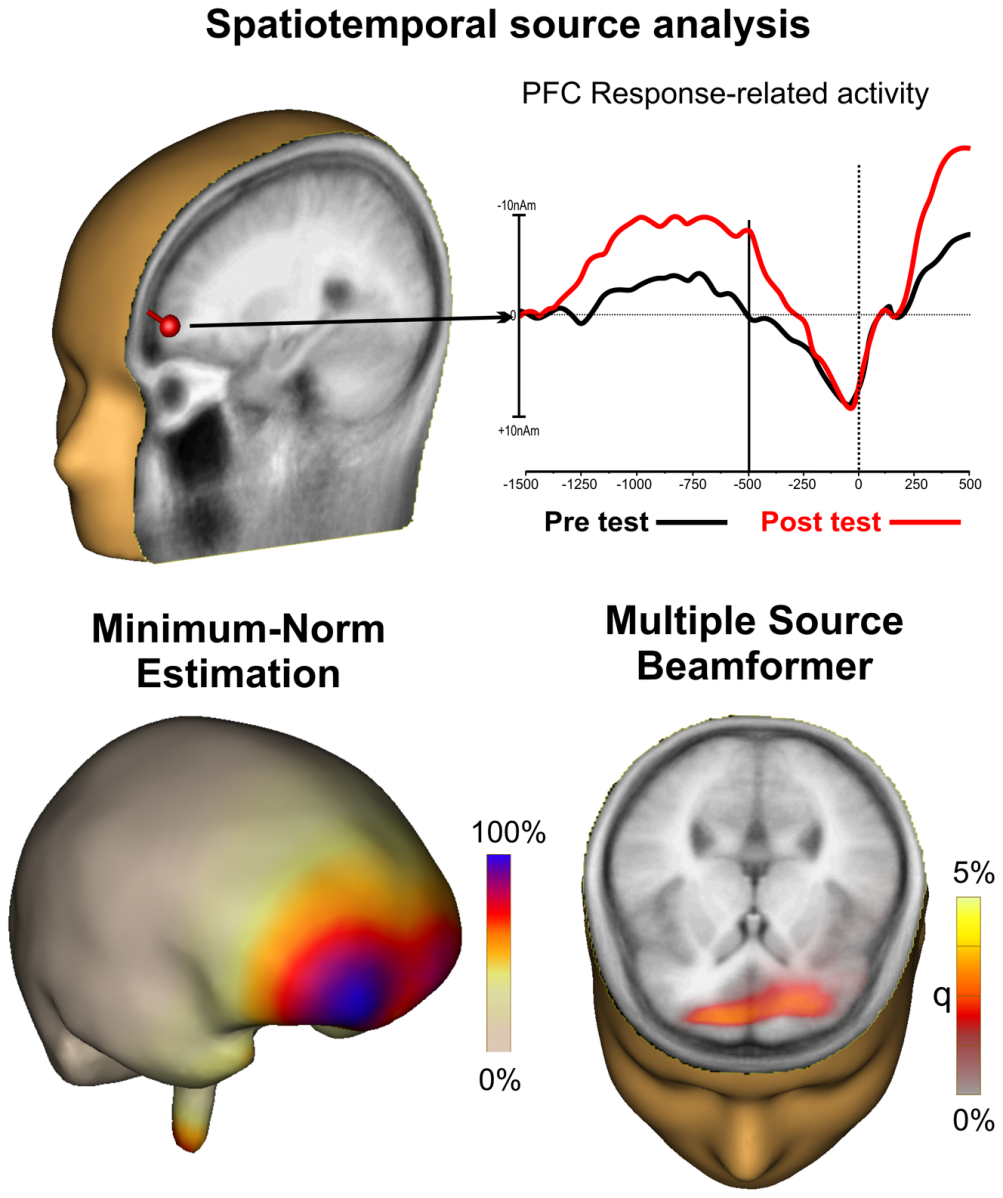
Representation of the spatiotemporal source analysis. Source analysis of the prefrontal activity using three different approaches yielded similar results.

Figure 6 shows the source localization of the MRCPs, indicating that the prefrontal activity arose bilaterally from Brodmann area 10 in the prefrontal pole of the PFC. Analysis of the MRCP source was employed using three independent techniques and all models yielded consistent results. Spatiotemporal source analysis is shown on top, indicating the dipole location and time-course (dipole moment) in the two conditions. MNE (lower left panel) showed the cortical surface activity, indicating a larger involvement of the right PFC, while the multiple source beamformer method (lower right panel) showed a more bilateral involvement, though the activity in the right hemisphere appeared more superficial than that in the left hemisphere.

## Discussion

Previous results have confirmed that the behavioral response and brain activity of MS patients are markedly deteriorated during visuo-motor discriminative response tasks [23], [24], [25]. The response time and PFC activityof the present patients (mean age 47 yrs) was comparable to those of healthy people in their eighties [12]. However, present findings show that a single one-hour HBP treatment improves visuo-motor response accuracy via compensatory mechanisms of the PFC. Indeed, the PCF compensatory activity, which seems stimulated by the HBP, interjects during both preparatory and executive phases of motor control, likely making more resources available to improve task accuracy, posture and gait.At the level of the PFC, the effect of the HBP on MS patients is the opposite of that played by physical activity on older people. In a previous work [12], we concluded that physical exercise reduces the age-related need of PFC compensatory activity, which is crucial to maintain their actions accurate. In the present study, the HBP seems to further stimulate the already hyperactive PFC, allowing a better response accuracy and motor control.

In summary, the present results reveal that the HBP rehabilitation device may improve accuracy, walking and posture in MS patients, because it could act upon multi-sensory and motor controlprocessing. This exoskeleton, which is not a cure for MS, further stimulated the already hyperactive PFC, which is fundamental in motor control.

The limitation of this study is the lack of instrumental measure to assess proprioception, balance and posture, given the restricted time available. Moreover, other parameters like the speed should have been considered in walking evaluations. Indeed, some studies suggest that the mean walking speed is more sensitive than EDSS in MS patients evaluations [26], [27]. With this regard, future studies might strengthen our findings by employing these measures, extending the number of participants and lengthening both the intervention and follow-up (more days per week and several months of treatment).

If confirmed, the present data will help to improve the quality of life for many individuals with MS. Using the HBP, clinicians and rehabilitation specialists can reduce clinical exacerbations and hinder disease progression. Although the HBP is not a cure, the MS treatments should also include agents that treat specific symptoms, improving the patients' ability to perform daily life activities such as walking [28]. Furthermore, the use of this innovative tool would be relevant for security and health services, because it may allow large savings in welfare costs and could also permit patients to stay at home for the HBP treatment. Future studies might also apply the HBP on amyotrophic lateral sclerosis and patients to improve their dysexecutive syndrome and mobility [29].
